# An amateur's contribution to the design of Telford's Menai Suspension Bridge: a commentary on Gilbert (1826) ‘On the mathematical theory of suspension bridges’

**DOI:** 10.1098/rsta.2014.0346

**Published:** 2015-04-13

**Authors:** C. R. Calladine

**Affiliations:** Department of Engineering, University of Cambridge, Cambridge CB2 1PZ, UK

**Keywords:** suspension bridge, Menai Bridge, Davies Gilbert, Thomas Telford, catenary, catenary of equal strength

## Abstract

Davies Gilbert's work on the catenary is notable on two counts. First, it influenced Thomas Telford in formulating his final design for the Menai Strait suspension bridge (1826); and second, it established for the first time the form of the ‘catenary of equal strength’. The classical catenary is a uniform flexible chain or cable hanging freely under gravity between supports. The ‘catenary of equal strength’ is the form of a cable whose cross-sectional area is made proportional to the tension at each point, so that the tensile stress is uniform throughout. In this paper I provide a sketch of the lives and achievements of Gilbert and Telford, and of their interaction over the Menai Bridge. There follows a commentary on Gilbert's 1826 paper, and on his two related publications; and a brief sketch of the earlier history of the catenary. I then describe the development of the suspension bridge up to the present time. Finally, I discuss relations between mathematical analysts and practical engineers. This commentary was written to celebrate the 350th anniversary of the journal *Philosophical Transactions of the Royal Society*.

## 1. Introduction

Davies Gilbert begins his 1826 paper [1] by explaining how, when Thomas Telford was working on the design of the Menai Strait suspension bridge in 1821—a bridge that was to have the world's longest span when it opened in 1826—he persuaded Telford to increase the height of the towers above the roadway from 25 to 50 ft,^1^ in order to ‘ensure the bridge's strength and permanence’. Telford, then aged 65, was an enormously experienced and inventive civil engineer. By contrast, Gilbert, 11 years younger, was a politician: a Cornish member of parliament since 1804, and a member of the Parliamentary Commission which was behind the funding of the Menai Bridge project. He had read mathematics at Pembroke College, Oxford.

It was hardly usual for an amateur client to persuade a seasoned engineer to make a major change to the design of an important and novel structure. Gilbert achieved this feat by publishing a short paper in the *Quarterly Journal of Science*, 1821 [2], in which he analysed the form of the *catenary* (Latin: *catena*=chain)—a heavy uniform flexible chain hanging freely under gravity between supports. He showed that the greatest tension in the chain occurred at the points of support; and that, for a given horizontal span between the supports, the greatest tension would be minimum if the vertical dip of the chain at its centre were approximately 0.34 or 1/3 of the span. By contrast, Telford was proposing for the bridge a dip of 25 ft in the suspension chains over a span of 560 ft; a ratio of 25/560=0.045 or 1/22, giving a tension about four times larger than the theoretical minimum for the span. Gilbert showed that if the dip were to be doubled, to 50 ft, the tension in the chain would be halved: and this point was accepted by Telford.

In this paper I shall give a sketch of Gilbert's career, and of the Parliamentary Commission to improve road communication between London and Holyhead. Then I shall describe briefly Telford's work on the design of the Menai Bridge. Next, I shall discuss in detail Gilbert's three papers on suspension bridges and catenaries: those of 1821 and 1826, already mentioned, and a later one in *Philosophical Transactions* of 1831 [3].

The paper of 1826 contains extensive tables for facilitating the practical design of suspension bridges. The paper is also distinguished for providing a completely novel analysis of the ‘catenary of equal strength’; that is, a catenary whose cross-sectional area is made to vary along its length to be everywhere proportional to the local tension, so as to achieve a *uniform (‘equal’) tensile stress* in the chain or cable. After this, I shall sketch the earlier mathematical history of the catenary, the subsequent history of the suspension bridge and the relationship between practical engineers and applied mechanicians in the 1820s.

The Menai Suspension Bridge is generally acknowledged to be an outstanding piece of civil engineering. But it swayed alarmingly in severe winds and had to be repaired in minor and major ways on several occasions, before Telford's original chains were replaced by stronger ones in 1940. Wind-induced vibration of suspension bridges of longer spans has been a permanent problem with this type of bridge; and in my sketch of the development of these bridges, I shall mention several different ways of dealing with the phenomenon.

## 2. Davies Gilbert (1767–1839) [4–6]

**Figure 1.**
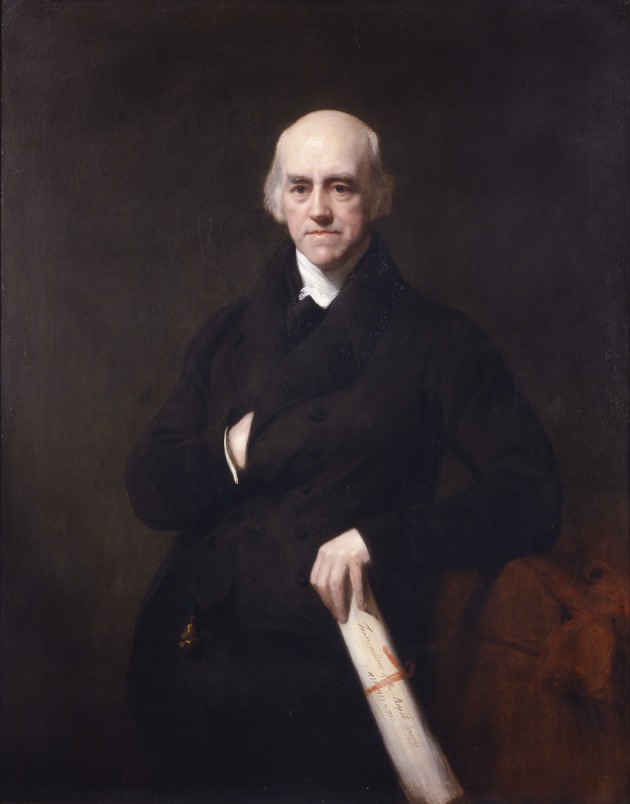
Portrait of Davies Gilbert. Copyright The Royal Society.

Davies Gilbert (figure 1) was born as Davies Giddy in 1767: his father, Edward Giddy, was curate of the parish of St Erth in Cornwall. At the age of 18 he entered Pembroke College, Oxford as a gentlemen commoner, where he studied mathematics, astronomy and other sciences. After graduating MA in 1789, Giddy began a decade of service to Cornwall, in maintenance of public order, preparation to repel invasion and control of food supplies. He served as high sheriff in 1792–1793 and was appointed deputy lieutenant in 1795.

He was elected FRS in 1791. His mathematical skills were deployed in helping Cornish engineers and inventors to develop more efficient steam engines: in particular, Hornblower with his compound steam engine and Richard Trevithick with his novel high-pressure steam engine.

Giddy was elected a member of parliament, first for Helston (1804–1806) and then for Bodmin (1806–1832). He refused office at Westminster under successive ministries, but instead served on and chaired numerous parliamentary committees, dealing with legislation on commodity prices, public works and weights and measures. Several of his committees were concerned with scientific and technical matters, such as the establishment of an astronomical observatory at the Cape of Good Hope, and the funding of Charles Babbage's Difference Engine.

In 1808, at the age of 41, Giddy married Mary Ann Gilbert, heir to her uncle's substantial estates in Sussex. These passed to Giddy on the death of the uncle in 1814, with the condition that the name of Gilbert be perpetuated. Thus, Davies Giddy became Davies Gilbert in 1817.

Gilbert's tireless parliamentary efforts on behalf of science, and his service to the Royal Society, persuaded Sir Joseph Banks (1744–1820), the society's long-serving and conservative president, to appoint Gilbert as one of its vice presidents in 1819 and to nominate him as his successor when he became ill in 1820. But reform was in the air, and Sir Humphrey Davy was elected president at the Anniversary meeting in November 1820, with Gilbert as treasurer. (It was at this time that Gilbert was a Holyhead Road Commissioner and was investigating catenaries.) In 1827 Davy resigned the presidency on account of ill health, and Gilbert became president for 3 years, after having failed to interest Sir Robert Peel (1788–1850) in the position. The Society was divided on the question of reform of its administration after the long reign of Banks, and there was much in-fighting among the fellows. The Duke of Sussex, a younger brother of King George IV, was admitted as a royal fellow in 1828; and Gilbert engineered his election as president in 1830, amid cries of ‘borough-mongering’ from the reformers. During this period of agitation for reform, the desirability for fellows to contribute to *Philosophical Transactions*, rather than to belong to ‘the class of absolutely inactive members’ was stressed. It is perhaps significant that Gilbert's 1826 paper was the first of five that he contributed to the journal [6].

## 3. Thomas Telford (1757–1834) and the Menai Bridge [7–10]

In 1820 Thomas Telford was Britain's leading civil engineer. He had widespread experience in the construction of roads, bridges, canals and docks. He had been involved in setting up the Institution of Civil Engineers; and he was now its first president, a position that he held until his death in 1834.

Telford was the son of an impoverished Scottish shepherd. On leaving school at the age of 15, he was apprenticed to a local stonemason. In 1780 he moved to Edinburgh in order to gain experience and to study the local architecture; and by the age of 25 he was working as a stonemason on Somerset House in London. Two years later, he was superintending the building of the dockyard commissioner's house at Portsmouth. Soon after that, with a reference from Robert Adam (1728–1792), he was operating as Shropshire's county surveyor of public works, directing work on public buildings and at least 42 masonry bridges. By 1790 Telford was advising on the improvement of numerous harbours and settlements in northern Scotland; and a few years later he was in charge of constructing the 68-mile Ellesmere Canal, linking the rivers Mersey, Dee and Severn. He also constructed several large-span masonry bridges over the Severn. Telford was involved in the construction of many new or improved roads in the highlands, with numerous masonry bridges. These major roads were generously built with gentle gradients, good foundations and good drainage.

Telford was an innovator in using iron for bridge building; and in 1800 he made a very bold proposal for a 600 ft span cast-iron arch bridge over the Thames, to replace London Bridge. He consulted widely on this novel project, as was his normal custom.

Telford became involved in the Menai Bridge project in 1810; but we need to go back 35 years before then, in order to understand how the project developed.

The Menai Strait separates the Welsh mainland from the Isle of Anglesey—from where, at Holyhead, ships sail to Ireland. Traffic to and from Ireland had to cross the strait by ferry; which was hazardous in the frequently stormy weather. Building a bridge over the strait came within the bounds of possibility in the late eighteenth century; and the question was raised in Parliament in 1775. After the Union between Ireland and the United Kingdom in 1801, Irish traffic became more important; and the Secretary of State for Ireland instructed John Rennie (1761–1821) [11] to prepare plans for a bridge. Rennie produced two designs, with a cast-iron arch of span 450 ft and rise of 150 ft; but he was doubtful of its practical execution. The height of the arch reflected the need to allow the passage of tall ships. The estimated cost of £260 000 was expensive; there was a war with Napoleon; and the project was shelved.

In 1810 the Government gave instructions to Telford to prepare a new scheme. The road between London and Holyhead needed much improvement, and Rennie disdained to work on roads. Telford also proposed a cast-iron arch bridge (span and rise 500 and 100 ft, respectively) at about half the cost of Rennie's scheme; and he devised a method of construction in which wooden centring for the arch would be supported by wrought-iron bars radiating from temporary wooden towers erected on the masonry abutments.

By an Act of 1815 the Government appointed Commissioners with powers to execute improvements to the Holyhead route; and the Commissioners in turn appointed Telford as engineer, and asked him to design a suspension bridge. Telford appointed William Provis (1792–1870) as resident engineer for the project.

**Figure 2.**
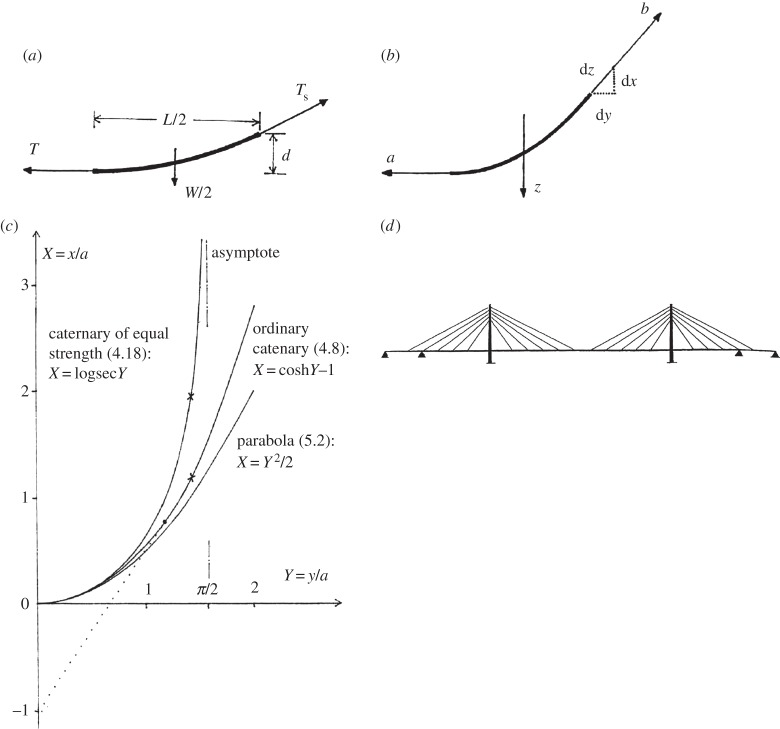
(*a*) ‘Free’ portion of a uniform half-chain. The tension is *T* at the lowest point, and *T*_s_ at the support. The approximate equation (3.1) is obtained by taking moments of the forces *T* and *W*/2 about the support: the equation is approximate because the centre of gravity of the curved half-cable is assumed to be *L*/4 from the support. (*b*) ’Free’ portion of a half-chain, from which, by statics, equation (4.1). The force *z* acts at the centroid: *z* is the arc-length of the curve. (*c*) Plot of three curves (4.8), (4.18), (5.2), as marked. The limits of Gilbert's tables are marked by cross symbols (×). Dotted tangent to (4.8) and filled circle (•) indicate the special point investigated by Gilbert in [2]. (*d*) Schematic layout of a typical cable-stayed bridge [13].

Telford had become involved, in 1814, with an ambitious speculative project for a suspension bridge over the Mersey at Runcorn, having a central span of 1000 ft and two side-spans of 500 ft. His general approach to innovative design was through experiment. Thus, he first measured the tensile strength of wrought iron from different sources (see the appendix of the book by Peter Barlow (1776–1852) [12]). Then, using wrought-iron wire suspended over various spans and with different degrees of central dip, he determined the load-carrying capacity. In an important test, he suspended a 7/8 in. square bar over a span of 125 ft and measured the tensile forces required to reduce the central dip to different fractions of the span. The results [9] were consistent with a broad general rule
3.1
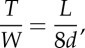

where *W* is the total weight supported before failure, *T* is the tension in the wire, *L* is the span and *d* is the dip of the cable at the centre. (Such an expression would today be obtained by an elementary consideration of the statical equilibrium of a ‘free body’ consisting of half of the cable (figure 2*a*) by a balance of the moments of forces *T* and *W*/2 about the point of suspension. Statics was evidently an uncertain art among practical engineers in those days; and in particular, the usefulness of ‘moment equilibrium’ was apparently unknown to Gilbert and his contemporaries. It is also noteworthy that all of those concerned with the design of the Menai Bridge used the terms ‘strain’ and ‘power’ as alternatives to ‘force’ and ‘tension’.)

The design of the Runcorn Bridge went through a number of revisions, aimed at encouraging more plentiful private financial investment. That never reached an adequate level; and so the project eventually failed.

During this period of discussion over the Runcorn Bridge, Telford met Samuel Brown (1776–1852) [14], a naval officer who made novel wrought-iron *chain anchor-cables* for ships, at his works on the Isle of Dogs. Brown had also patented a design for wrought-iron eye-bar links for use in suspension bridges; and he had made a machine for testing the strength of chains and links in tension. In evidence to the Select Committee of the House of Commons [15], Rennie described how he had been drawn in a carriage over a small-scale suspension bridge of span 120 ft at Brown's works, and found himself ‘perfectly safe and easy’.

Telford was so impressed by Brown's work that he decided to use eye-bar links for the Menai Bridge, in preference to his previous scheme for cables made up from bundles of half-inch square wrought-iron rods. Maintenance, and protection against corrosion, would be under better control with eye-bar links.

Brown used eye-bar chain for the Union suspension bridge across the Tweed near Berwick, which he built in 1820 in collaboration with Rennie, who designed the masonry: it had a span of 450 ft and, after modification, is still in use today.

In the course of his work on the Menai Bridge, Telford made numerous changes to the design: there were no precedents to guide him, and he proceeded with extreme caution; see Paxton [9] for a full account of the evolution of the Menai design. Indeed, many changes were made after the design had been approved by the Commissioners in 1818 [16] and by the Select Committee in April 1819 [15]; and an Act of Parliament had been secured in July 1819. Thus, Telford increased the span to 580 ft and adopted chain links. On the advice of Rennie, he increased the cross-sectional area of the chains; and, as we have seen, he increased the dip of the chains on the advice of Gilbert. He also replaced the previous cast-iron ‘pyramids’ by towers of dowelled masonry, and moved the anchorages of the chains into deep tunnels in the rock.

John Provis (1801–185?), younger brother of William, was Telford's colleague in charge of the ironwork of the bridge, and he tested 15 000 eye-bar links and many other components to a tensile stress of 11 ton in^−2^—about twice the design working stress.^2^ Each of the 16 chains—making four groups, each four chains deep—was hauled into place by capstans powered by a team of 150 men, working in shifts.

**Figure 3.**
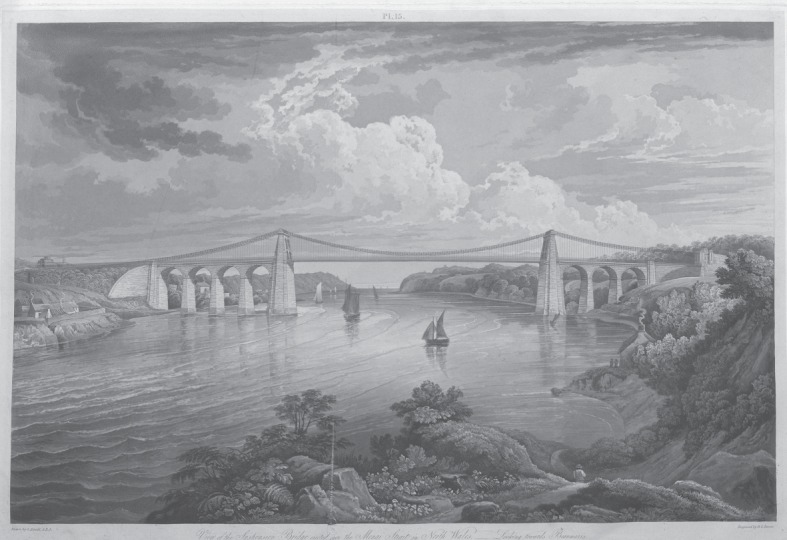
The Menai Suspension Bridge; from the book by William Provis [17]. Drawn by G. Arnold and engraved by R. G. Reeve. Courtesy of the Institution of Civil Engineers.

The bridge (figure 3) was opened in January 1826. Its total cost was £232 000, which was three times the original estimate. (When further funds were being sought in 1823, it was stated that ‘Although the Commissioners are disposed to agree in opinion with Mr Telford, that a bridge of slighter construction would have been perfectly secure, yet a greater degree of strength having been recommended by Mr Davies Gilbert, Mr Rennie and Mr Barlow, they felt no hesitation in complying with the suggestions of persons of so high authority’ [18].)

The bridge remains an outstanding engineering achievement, and has outlasted other bridges of its time. In 1832 William Provis published a large-format book [17], copiously illustrated, to describe the entire history of the project, including a thorough description of construction and its problems.

## 4. Gilbert's catenary papers

Gilbert's papers of 1821 and 1826 both contain essentially the same analysis of the ‘ordinary’ catenary; so I shall discus their contents together.

**Figure 4.**
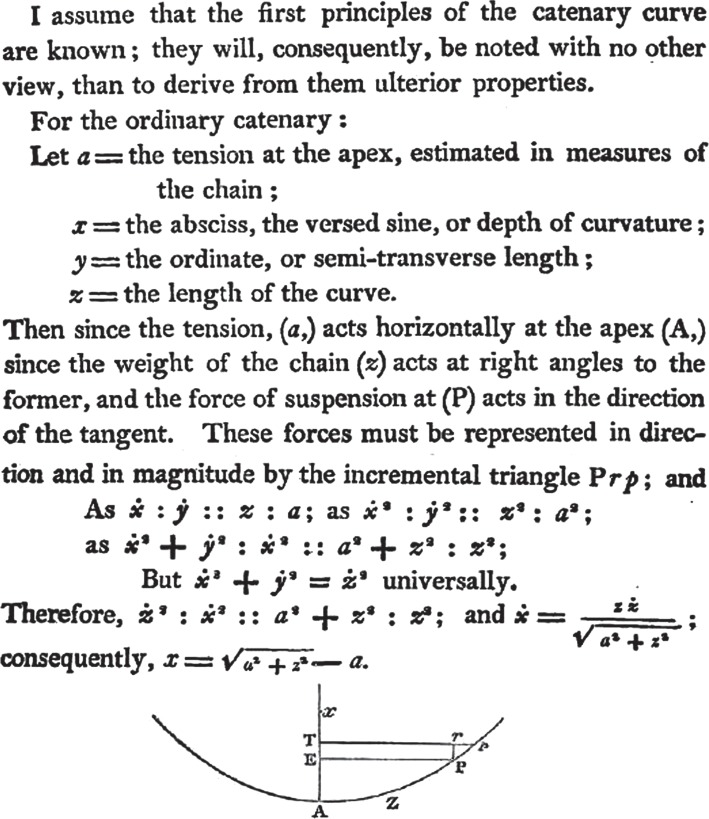
A portion of Gilbert's text [1], assembled from pages 203, 204 and 212. The *y*-axis, not shown, goes to the right from point A. Copyright The Royal Society.

Figure 4 shows the opening section of the working, as it appears in 1826. His tension *a* at the lowest point (‘the apex’) ‘is estimated in measures of the chain’; i.e. it is equal to the weight of length *a* of chain.

In my commentary, I shall stick to Gilbert's use of *x* and *y* to denote vertical and horizontal components of position of a general point on the curve, rather than today's normally opposite usage; and to his use of *z* for distance measured along the curve from the origin to a general point, rather than arc-length *s*, as is usual today. But I shall denote infinitesimals by d*x*, d*y*, d*z* rather than by Gilbert's 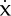, 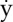, etc. after the usage of Newton.

By considering the statical equilibrium of a general portion—a ‘free body’—of the chain (figure 2*b*), and noting that the horizontal tension *a* at the lowest point and vertical weight *z* are balanced by a tension *b* according to a triangle of forces, and that the slope of the tensile force is equal to d*x*/d*y*, Gilbert obtains this differential equation for the curve:
4.1
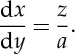

Noting that
4.2
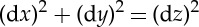

by Pythagoras' theorem, he eliminates d*y* to obtain an expression for d*x*/d*z* as a function of *z*. Integrating, and using the condition that *x* and *z* both vanish at the origin (which he places at the lowest point of the chain), he obtains
4.3
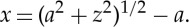

Also, by the triangle of forces
4.4
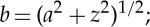

hence
4.5
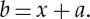

Thus, the tension in the chain increases directly with *x*, from its value of *a* at the origin. Using (4.1) and (4.3) together, Gilbert obtains
4.6
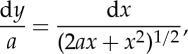

and by integration—again with the condition that *x* and *y* both vanish at the origin
4.7
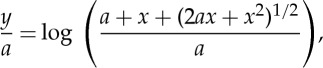

where log stands for ‘natural logarithm’—or hL, as Gilbert writes, for the ‘hyperbolic logarithm’.

In the 1826 paper, Gilbert goes on to manipulate (4.7) in order to get *x*/*a* explicitly as a function of *y*/*a*. His final expression is exactly equivalent to the well-known modern formula (when the origin is fixed at the lowest point of the curve) (see figure 2*c*)
4.8
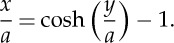



Later, when Provis included the 1826 paper as Appendix 9 of his great book [17], Gilbert missed the opportunity to correct a typographical error in the original, but he added a page of tidying-up to give (4.8) explicitly, though in terms of the exponential function e^*y*/*a*^: he may not have been aware of the functions sinh and cosh, which had been introduced in the mid-eighteenth century.

At this stage in the 1821 paper, Gilbert goes on to work out the condition for d*b*/*b*=d*y*/*y*. Although he does not say so explicitly, this is equivalent to finding the condition for the point at which a line from point (*x*,*y*)=(−*a*,0) touches the curve: see figure 2*c*. And, as may readily be shown, this is tantamount to the condition that the tension in the chain at the points of support, for supports separated by a given span, is as small as possible.

He obtains this condition by means of a numerical process of trial-and-error; and he finds *x*/*y*=0.81/1.2, or *d*/*L*=0.34 where, as before, *L* is the span and *d* is the dip. For a span of 560 ft, the minimum tension at the supports, *b*, is 418 ft. He then points out that this optimum geometry, with such tall towers (190 ft for a span of 560 ft) would be impractical for a real bridge.

Gilbert then (1821) turns his attention to the dimensions being proposed by Telford for the Menai Bridge: *L*=560 ft, *d*=25 ft. He realizes that for such a small value of *d*/*L*, and hence of *x*/*a*, the natural logarithm in (4.7) may be simplified by a series expansion; and in this way he finds the following dimensions, all in feet: *y*=560/2=280, *x*=25, *z*=282.2, *a*=1580 and *b*=1605. Thus the peak tension *b* in the chain at the support is almost four times larger than for the optimized chain. Finally, Gilbert works out the case where *x* is doubled to 50 ft: *y*=280, *x*=50, *z*=288, *a*=808 and *b*=858. Here the values of both *a* and *b* are approximately half of those above. That is the point which evidently impressed Telford: the larger the dip of the chains, the smaller the tension in them, other things being equal—and indeed just as in (3.1).

The portion of the 1826 paper concerning the ‘ordinary catenary’ concludes with two large tables, designed for practical use by those involved in the design of ‘bridges of suspension’. Gilbert explains that the catenary is a universal curve—like the circle, the parabola, the logarithmic curve, etc.—which can be enlarged or scaled in order to apply to any particular case; and here the key scaling parameter is *a*, the tension in the chain at its lowest point.

Thus, Table II is constructed for *a*=100; it has six columns headed, respectively, *N* (=*e*^*y*/*a*^), *y*, *x*, *z*, *T* and Angle (of the tangent at the support, measured from the vertical). The table has 100 rows, for *y*=1,2,3,…100. (In this (1826) paper, the chain's tension at the supports is denoted by *T*, rather than by *b*, as in the earlier paper.) Apart from *y*, all numbers are given to six places of decimals, while angles are in degrees, minutes and seconds. The *y*, *x* values give the form of the catenary (4.8) shown in figure 2*c*; and Gilbert explains in detail how to use the table for other values of the parameter *a*.

Given that Gilbert has explained how to use this universal Table II, it is perhaps surprising that he should also provide Table I, for which *y*=100 (i.e. for a span of 200 units). Here the columns are headed, respectively, *a*, *N*, *x*, *z*, *T* and Angle. He points out that Table I shows explicitly that, for a given span, the tension at the supports does indeed fall to a minimum, as shown in the 1821 paper; and the table continues for a few lines beyond that point.

The second part of this 1826 paper deals with the *catenary of equal strength*, which is generally acknowledged to be Gilbert's genuinely original, ‘landmark’, contribution to catenary studies. As we have seen, the tension in the ‘ordinary catenary’ increases directly with height *x*. In Tables I and II, for instance, there are catenaries in which the tension at the support is 50% larger than at the lowest point. In such a case, it would clearly be inefficient to size the (uniform) chain in accordance with its peak tension. (We recall that Gilbert had previously been concerned with calculation of the efficiency of steam engines.)

Thus, Gilbert conceived the idea of a chain whose strength, locally, matched the tension. Here it is easier to think in terms of a smooth, flexible cable, made from a material of given specific gravity, whose cross-sectional area is proportional to the local tension.

Gilbert tackled this problem by introducing a new variable, *ζ*, to denote the total mass of the chain from its lowest point to a general point under consideration, in addition to the variables *a*, *x*, *y*, *z*, as before. The force *ζ* is expressed in terms of the length of cable whose cross section is that of the cable at its lowest point.

By overall equilibrium of a free body, (4.1) is now replaced by
4.9
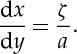

Eliminating d*y* between (4.9) and (4.2), we have
4.10
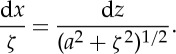

Also, the local tension, *T*, is given by the triangle of forces
4.11
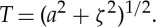

Next, Gilbert states, laconically: ‘on the principle of equal strength
4.12
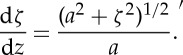

This step warrants some explanation. d*ζ*/d*z* is the local weight per unit length of cable; which, for a given material, is proportional to the cable's cross-sectional area; which in turn is proportional to the local tension, if the tensile stress is to be uniform. That explains the numerator on the right of (4.12). Now our method of expressing force as the weight of a length of chain—both for the ‘ordinary catenary’ and here at the origin—implies a weight per unit length of unity; hence the denominator on the right.

Rearranging (4.12), integrating and noting that both *ζ* and *z* vanish at the origin:
4.13
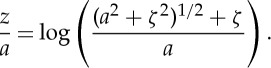

Combining (4.10) and (4.12), we get
4.14
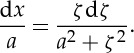

Integrating, we find
4.15
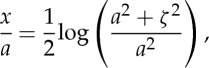

after using the condition that both *x* and *ζ* vanish at the origin.

Combining (4.9) and (4.14), we have
4.16
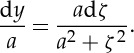

Integrating, and noting that both *y* and *ζ* vanish at the origin:
4.17
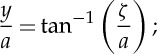

or, as Gilbert puts it, in those days before trigonometric functions were commonly expressed as ratios: ‘*y*=the circular arc of which *ζ* is the tangent to radius *a*’.

Gilbert now constructs his Tables III and IV as follows. First, he takes pairs of values of *y* and *a*; next, he uses (4.17) to obtain *ζ*; then (4.15) and (4.13) provide *x* and *z*, respectively; and finally, he uses (4.11) to get *T*. For Table III, *y*=100 and the column headings are: *a*, *x*, *z*, *ζ*, *T* and Angle; while for Table IV, *a*=100 and the column headings are: *y*, *x*, *z*, *ζ*, *T* and Angle.

This curve of *x*/*a* against *y*/*a* is also shown in figure 2*c*. As Gilbert points out, equation (4.17) immediately tells us that *y*/*a* cannot exceed *π*/2. As the curve approaches its vertical asymptote, the cross-sectional area of the cable increases exponentially with height, just as in a vertical, constant-stress cable, suspended from above.

Later workers, such as Routh [19], have produced more elegant solutions of this problem, principally by using tension *T* and the slope *ψ* of the tangent to the curve as variables. This produces an explicit relation between *x* and *y*
4.18
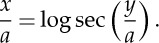

And indeed another very simple result emerges:
4.19
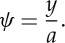

This equation would enable the curve to be extended readily from the origin by graphical or computer-stepping procedures; and, of course, the profile of the ‘ordinary catenary’ could be constructed likewise from its governing equation (4.1).

Gilbert finishes the paper with some practical remarks, ‘wholly unconnected with the preceding investigations’. To ‘counteract and restrain undulatory motion’—occasioned, we suppose, by wind forces or by the passage of a heavy load—Gilbert recommends that ‘the balustrades may be carried to any required height and rendered inflexible by diagonal braces’. In other words, he foresees the need to stiffen the bridge by means of a *roadway* *girder*. Quantitative analysis of the stiffness of such girders, and of interaction between them and the property of suspension chains to resist perturbations of their ideal geometry, did not emerge until the work of Rankine [20] in 1858 (see Pugsley [21]).

Gilbert's third catenary paper, of 1831, is very short, and it makes no mention of the catenary of equal strength. He explains, in effect, that his previous tables would have been of more practical use if data for specific values of *d*/*L* had been tabulated in the range 1/40≤*d*/*L*≤1/7 of practical suspension bridges.

The new table has five columns. The first is headed ‘Deflection or versed sines’, and it contains values of both *L*/*d* and *d*/*L*. (At this time, the term *versed sine* was used widely to denote the dip or ‘deflection’ of a suspended cable. It is defined as the largest separation between a (nominally circular) arc and its chord; and it is sometimes called *sagitta* (Latin) or *flêche* (French) after the bow-and-arrow analogy.) The second column, ‘Length of the chains’, gives the length of chain divided by the span. The third and fourth, ‘Tensions at the middle points’ and ‘Tensions at the extremities’, respectively, give tensions as multiples of the total weight of the chain. The fifth gives ‘Angles with the horizon at the extremities’. Each of the 52 rows is adapted ‘by simple arithmetric’ from a row of Table I of the 1826 paper. There are now fewer significant figures, and angles are given only in degrees and minutes. This compact table is clearly of more immediate use to engineers than those of the 1826 paper. I shall comment below on the accuracy of simple formulae based on the use of *parabolas* rather than catenaries.

## 5. Follow-up to Gilbert's work

The form adopted by a chain hanging under gravity is not necessarily directly relevant to the design of a suspension bridge. For example, we could have a bridge of small span, with a light cable supporting a heavy horizontal roadway. Instead of governing equation (4.1), we would then have
5.1
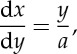

as the vertical loading would be uniform along the horizontal axis; and by integration
5.2
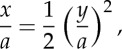

when the origin is put, as usual, at the lowest point of the cable. The parabola of equation (5.2) has also been plotted in figure 2*c*; and it is clear that it provides a fair approximation to the curves of the two types of catenary (4.8), (4.18) when the values of *d*/*L* are not larger than, say, 1/10.

Moseley (1801–1872) [22] was concerned that, in general, the chain or cable of a suspension bridge must support both its own weight (which is uniform along the contour of the cable) and the weight of the (horizontally uniform) roadway; and he produced complicated analytical solutions to the appropriate governing differential equation. He further complicated matters by putting in the extra weight of the vertical ‘hangers’, in the space between the cable and the roadway.

Rankine (1820–1872) [20], who combined mathematical dexterity with the common sense of a practical engineer, pointed out that for all values of *d*/*L* relevant to actual bridges, Moseley's complicated formulae demonstrated that there was no need to use anything more elaborate than a parabola for the form of the cable.

Experience with the behaviour of the Menai Bridge in strong winds quickly drew attention to the desirability—pointed out by Gilbert, as we have seen—of having the roadway girder sufficiently stiff to moderate undulations of the chains on account of the wind or heavy localized loading on the roadway. Rankine was the first to propose a simple way of fixing the flexural stiffness of the roadway girder. Pugsley [21] has given a clear description of subsequent developments in this important area of design. A key point here is that the cable itself responds to perturbations of loading by perturbations in curvature; and the stiffness of this geometry-change-effect is proportional to the cable's tension for a given value of *d*/*L*. That, in turn, is broadly proportional to the span; and so the problem becomes less severe for larger spans.

## 6. Earlier studies of the catenary [23–29]

Robert Hooke (1635–1703) made a crucial observation for the design of masonry arches. In 1676 he pointed out that ‘as hangs the flexible line, so but inverted will stand the rigid arch’ [23]. Thus, if such a curve could be drawn within the voussoirs of a masonry arch, that arch would stand. This brilliant piece of intuitive thinking is the basis of rational design of all masonry arches, vaults and domes [26–28]. (Hooke put forward his theorem in the form of an anagram; that was an extreme ploy in the seventeenth century for claiming priority for a result without revealing the working behind it.)

Hooke used this method for the design, with Wren (1632–1676), of the great dome of St Paul's Cathedral in London: the weights of a radial ‘slice’ of masonry blocks were hung from an imaginary light chain for this purpose. St Paul's is the only large masonry dome which has not shown signs of distress by cracking. (The apparently cylindrical drum above the ‘whispering gallery’ is in fact slightly conical, in accordance with the shape of the inverted catenary.)

In 1673 I.-G. Pardies (1636–1673) published *La Statique*in Paris. He claimed that the catenary curve would be unchanged if any parts of it were solidified. He also stated that if portions of a chain were removed, conceptually, they could be replaced by suitable forces, tangential to the curve, acting on the remaining portions. In other words, he proposed the idea of considering the statical equilibrium of forces acting on a ‘free body’.

In May 1690 Jacob Bernoulli (1654–1705) proposed, in *Acta Eruditorum*, Leipzig—in Latin—a contest to find the catenary curve. In June 1691 the same journal published three solutions, by Johann Bernoulli (1667–1748), Gottfried Leibniz (1646–1716) and Christiaan Huygens (1629–1693). Of these, only Leibniz gave an equation, equivalent to (4.8), but without proof and with no mention of the underlying differential equation; but he does state that his *differential calculus* was the key to the solution.

David Gregory (1659–1708), a Scottish mathematician at the University of Oxford, was keen to apply ‘the method of fluxions’ of his contemporary Isaac Newton (1642–1727) to the solution of problems. In 1697 he published in *Philosophical Transactions*—in Latin—an elaborate demonstration of Leibniz' result [29]. Unfortunately, he gave an erroneous derivation of the correct differential equation. Gregory's major error lay in his failure to appreciate that it is the *difference* between tensions on each end of a small element of chain which balances its gravity: as Truesdell [23] remarks, this is ‘one more example to show that the local balance of forces, which we are all taught to regard as the simplest approach to the mechanics of continuous media, is in fact not an obvious concept’. But once he has stated the correct equation, Gregory goes on to prove all of Leibniz' results competently. Gregory's paper was reprinted in *Acta*, July 1698. Leibniz soon pointed out, anonymously, Gregory's basic deficiency in the *Acta* of February 1699, unjustly attributing it to a defect in Newton's fluxional method. He challenged Gregory to discuss the problem with Newton; but Newton diplomatically declined to become involved. Jacob Bernoulli likewise attacked Gregory: ‘his work shows neatly how it is possible for us to be misled through an inevident and false, though plausible, argument to a true conclusion’.

## 7. A brief history of suspension bridges [13,21,30–38]

Joseph Needham [30] points out that the suspension bridge is the *sine qua non* for intercourse in historical times between people of China and those of Tibet, Afghanistan, Kashmir, Nepal, India, Burma and Thailand; and that the Chinese equivalent of Hindu Kush means ‘suspended crossings or passages’. Such bridges, for pedestrians, were supported by cables about 2 in. thick, plaited from bamboo strips. Similar bridges in the Andes [31] were essential for communication throughout the Inka Empire; and there the ropes were made from twisted grass, in periodic communal activity.

In China, bamboo cables were replaced by wrought-iron chains not later than the sixth century. Needham describes, for example [30, fig. 852], a bridge of span 225 ft in Yunan province, across the Mekong river on the old road to Burma: it has had iron chains since 1470; 12 chains under the roadway and two for handrails.

The earliest iron-chain bridge in Europe was built in 1741 over the Tees near Middleton. This ‘Winch’ footbridge was 2 ft wide, with a path following the catenary. It collapsed in 1802.

The credit for the first iron suspension bridge with a level deck is usually accorded to James Finley (1756–1828), who built his first chain bridge of 70 ft span across Jacob's Creek, Pennsylvania, USA in 1801: the roadway was 13 ft wide. Many bridges of similar design were constructed in eastern USA over the next 20 years, including the Merrimac Bridge in Massachusetts (1810) with a span of 240 ft and roadway of width 30 ft. After refurbishment, it still stands.

As mentioned in §3, Brown built the ambitious Union Bridge over the Tweed in 1820. It was the first bridge to be built with wrought-iron eye-bars. Its span was 450 ft and the deck was 18 ft wide. Several other bridges with eye-bar chains were built in England in the following 50 years or so. For example, W. T. Clark (1783–1852) built the first Hammersmith (London) bridge, span 400 ft, in 1827 [32]. He made and wind-tested a model and devised a stiffening girder of longitudinal trussed railings. (This bridge was replaced in 1887 [9].) And I. K. Brunel (1806–1859) built the first Hungerford (London) bridge, span 680 ft, in 1845. Its chains were later used for the Clifton (Bristol) bridge, span 700 ft, built to Brunel's design in 1864.

In 1821 C.-L. Navier (1785–1836) was sent by the French government to study suspension bridges in England. He published a report in 1823 which led to the construction of many suspension bridges in France, notably those over the Rhone by Marc Seguin (1786–1875), using cables made up from many strands of drawn wrought-iron wire.

As we have seen, Telford's Menai Bridge was opened in January 1826. Within months, its light timber deck was damaged by a severe gale, and many of the wrought-iron suspenders, with cross section 1 in. square, were broken. In the summer of 1826 ‘transverse chain bracing’ was incorporated at four locations along the span, in order to hold the four groups of chains at fixed separations; and the maximum undulation was thereby reduced. But in January 1839 a hurricane-force gale completely wrecked the wooden deck: most of the outer suspenders were broken at their joint with the roadway, and the free outer chains clashed with the inner chains. William Provis, Telford's resident engineer, re-designed the deck, increasing its total weight from 620 to 750 tons. That deck remained until 1893, when Sir Benjamin Baker (1840–1907) provided a new steel deck weighing 1000 tons.

In the USA, suspension bridges of ever longer spans were built over major rivers. John A. Roebling (1806–1869) made his cables from multiple drawn-steel wires; and he built the first long-span railway bridge at Niagara Falls in 1855: it had a span of 821 ft and was stiffened both by wooden truss-girders 16 ft deep, and by inclined rods radiating from the towers. His most famous Brooklyn Bridge, completed in 1883 (by his son and daughter-in-law, after his death) has a span of 1600 ft, and it also is stiffened by a girder and diagonal cables.

Many major suspension bridges in the USA were built between the two World Wars along similar lines, but without the diagonal stiffening cables; various schemes for proportioning stiffening girders were developed (e.g. [21,13]).

The dramatic failure of the Tacoma Narrows Bridge in 1940, by means of wind-induced flexural-torsional ‘galloping’, raised important questions about wind effects on slender structures. The bridge deck had steel I-beam stiffening girders, which provided little torsional stiffness. The violence of its motions had increased dramatically when diagonal links, intended to prevent relative horizontal movement between the main cables and the centre of the roadway girder, failed [13].

After World War II several long-span suspension bridges were built in the UK. Sir Gilbert Roberts (1899–1978) introduced roadway girders in the form of hollow steel boxes with ‘aerodynamic’ cross sections; and their use has become widespread. Such girders have high torsional stiffness; and more stiffness was provided by having the hangers from the main cables inclined instead of vertical, thus providing a more rigid, triangulated cable network. But an unintended consequence of this arrangement was that the passage of heavy loads produced extra tension in the hangers, resulting in failure by fatigue; and only a few bridges have been built with inclined hangers.

Aerodynamic effects in modern long-span suspension bridges are sometimes countered by division of the roadway into two steel boxes, linked by members which provide a central air-gap. The extra width between the main cables also contributes towards stability.

Corrosion can be a major problem for the main cables of suspension bridges, if water cannot be excluded from the compacted wire bundles. A modern procedure is to enclose the cable with an air-tight membrane and to blow dry air from one end to the other. The frictional clamps which enable vertical hangers to be attached to inclined cables provide an obstacle to such arrangements. One way around this problem—at least, for small pedestrian bridges—is to encase the cable's tendons in a plastic tube and to fasten the hangers orthogonally to the cable, thereby avoiding the need for tight, frictional clamps [37].

Lastly, we should mention the unexpected phenomenon of ‘pedestrian-induced lateral vibration’ which appeared as soon as crowds crossed the Millennium footbridge over the Thames in London in 2000. These vibrations had the effect of synchronizing the gait of the passengers, thereby maintaining oscillations. The bridge was later made serviceable by the installation of multiple dampers.

Numerous long-span suspension bridges have now been built in many different countries. For example, eight have been constructed during the present century, with spans in the range 1100–1700 m: six of them are in China.

Since the 1950s, a different form, the *cable-stayed bridge*, has been developed. The early steps were taken with new bridges over the Rhine, where intermediate piers were replaced by a few diagonal stays to the towers. A popular form today, with many cable-stays, is sketched in figure 2*d* [13].

Cable-stayed bridges have strong advantages over suspension bridges in terms of construction. Thus, one tower can be built and its roadway units placed—each with a particular cable anchored back to the tower—before the second tower is begun. The cables, which are much less massive than those for a corresponding suspension bridge, can be purchased ready-made from companies that began as manufacturers of tendons for pre-stressed concrete. And if an individual cable should suffer from corrosion, it can be replaced individually. By contrast, the construction of the roadway of a conventional suspension bridge cannot be started until both towers and the main suspension cables have been finished; and the incremental strand-by-strand process of building up the main cables from tens of thousands of 5 mm diameter wires is time-consuming.

A problem with long individual cable-stays is that they tend to vibrate in the wind, and thus suffer from fatigue damage; and the problem is more severe in the presence of rain and ice. Schemes developed recently for damping the rotation at the end-fixtures of cables have been successful in reducing fatigue damage [38].

During the present century, about 80 cable-stayed bridges have been built in some 16 countries around the world: about 60% of them are in China.

## 8. Conclusion

Our discussion of Gilbert's paper and Telford's bridge has illustrated some important points about the relationship between the academic theory of structures on the one hand, and the construction of real bridges on the other.

First, as we have seen, the theory of the catenary is useful (if not absolutely necessary) for the construction of suspension bridges; but there is, of course, much more than this to building bridges. Thus, Telford had to address a myriad of practical problems concerning the design, manufacture and testing of the 15 000 wrought-iron eye-bars; the construction of the masonry towers; the design of the cradles (on rollers) where the chains pass over the towers; the anchorages of the chains at either end of the bridge; means for replacing individual iron links of the chains, should that become necessary; and so on: see Provis [17] and Paxton [9] for a full account.

Telford was grateful for Gilbert's work; but earlier he had asked Barlow [12] and James Jardine (1776–1858) [39, Appendix 12, p. 684] for theoretical calculations on the tension in a cable of given span and dip. Barlow computed one example, for *d*/*L*=1/20, using formulae from Poisson (1781–1840) [40] by a roundabout method. Jardine in 1821 gave Telford results for three more cases with larger values of *d*/*L*; but we have no record of his working. (Both Barlow and Jardine gave results in agreement with Gilbert's 1831 table.) Telford sought advice from many individuals: the profession of ‘consulting engineer’ had not then been invented.

In reading Gilbert's papers, we can see what heavy weather he made of his calculations. He did indeed obtain an explicit expression for the form of the ‘ordinary catenary’ by his second paper in 1826; but it was less compact and elegant than the formula of Leibniz, obtained 135 years earlier. Gilbert also did not obtain an explicit formula for the shape of the ‘catenary of equal strength’, although he justly receives credit for solving that novel and difficult problem.

As we have seen, Gilbert—and indeed others—dealt with ‘shallow’ cables, having smaller *d*/*L* values, by using series expansions of logarithmic functions. None of these workers appears to have been aware of the technique of moment equilibrium for obtaining a good approximation (3.1) to the tension of the lowest point of the cable—particularly if the approximation d*z*/d*x*=(1+0.5(d*x*/d*y*)^2^) (in Gilbert's notation) is used for calculating the contour length and centre of gravity of the half-chain. This, together with (4.5), enables most of the entries in Gilbert's table of 1831 to be established within 0.5%.

Telford was cautious about analytical results obtained solely by means of applied mechanics. For example, when Telford was asked by the Select Committee [15, p. 30] ‘Do not the theoretical calculations he [Barlow] has published correspond very much with the practical experiments you have just mentioned?’, Telford replied ‘Very nearly; I do not go from any theory, I proceed upon experiments’. It is conceivable that Telford was particularly cautious about theories that involved, for example, logarithmic functions. Perhaps he might have been more receptive to simpler procedures such as taking moments (3.1), which involve simple arithmetic?

Two points emerge here. First, as Truesdell [23] has remarked, ‘As often happens in the history of science, the simplest ideas are the hardest to achieve; simplicity does not come of itself, but must be created’. And although Newton had laid down the principles of statical equilibrium, which are essential for design of all engineering structures, the practical analytical tools for use by engineers were not widely understood in the 1820s. Indeed, for the construction of railway-bridge girders later in the nineteenth century, the intellectual power of James Clerk Maxwell (1831–1879) [41] was required to set out the rules for constructing rigid framework structures and for analysing the tensions in their respective members on account of arbitrary applied loads. Only since the development of the digital computer has it been possible for engineers confidently to design complex practical cable-based structures—provided always that unsuspected phenomena are not encountered.

Our two main characters have been accorded tangible memorials: Telford is the name of a town in central England, and Davies Gilbert is the name of a mountain in the north of Canada's Yukon Territory. Further, Telford's Menai Bridge is on the obverse of a British one-pound coin of 2005, while Gilbert is commended in the *Oxford Book of Carols* [42, p. xiii] as the editor of the first modern collection of traditional (Cornish) carols.

## Footnotes

1 Note on physical dimensions. I give dimensions in inches (in.), feet (ft) and miles; or in metres (m), according to the conventions of the time. Approximately, 1 in.=0.025 m; 1 ft=0.30 m; 1 mile=1600 m.

2 Note on units of stress. A typical value of breaking stress for wrought iron is 22 ton in^−2^=340 MPa; 1 pascal (Pa)=1 N m^−2^.

## Author profile

**Chris Calladine** is emeritus Professor of Structural Mechanics in the University of Cambridge. Most of his career has been spent in the Department of Engineering there, teaching and thinking about phenomena and puzzles in structural engineering. His early work was on creep and buckling of pressure vessels and fuel-element cladding in nuclear power reactors. Later he studied the performance, including buckling, of thin-walled shell structures in general; and he clarified the key role of Gaussian curvature in understanding the subject. He also built on Maxwell's work on special situations of anomalous behaviour in structural assemblies of rods and joints, which has led to better understanding of membrane and deployable structures. He has also collaborated with biologists in elucidating the behaviour of some biological structures on a molecular scale, particularly DNA and the polymorphic corkscrew-like flagellar filaments that act as bacterial propellers. These structures are helical; and indeed the flagella have a hierarchy of four levels of helical structure. In both cases bi-stability of structural components is an important feature of the performance.
